# The effect of common variants in GDF5 gene on the susceptibility to chronic postsurgical pain

**DOI:** 10.1186/s13018-021-02549-5

**Published:** 2021-07-01

**Authors:** Shaoyao Yan, Huiyong Nie, Gang Bu, Weili Yuan, Suoliang Wang

**Affiliations:** Department of Pain, The First Affiliated Hospital of Xi’an Jiaotong University, No. 277, Yanta West Road, Xi’an, Shaanxi China

**Keywords:** Chronic postsurgical pain, Single nucleotide polymorphism, *GDF5* gene, Case-control study

## Abstract

**Background:**

The growth differentiation factor 5 (GDF5) gene regulates the growth of neuronal axons and dendrites and plays a role in the inflammatory response and tissue damage. The gene may also be associated with chronic postsurgical pain. This study aimed to reveal the relationship between SNPs in the *GDF5* gene and orthopedic chronic postsurgical pain in Han Chinese population based on a case-control study.

**Methods:**

We genotyped 8 SNPs within *GDF5* gene in 1048 surgical patients with chronic postsurgical pain as the case group and 2062 surgical patients who were pain free as the control group. SNP and haplotypic analyses were performed, and stratified analyses were conducted to determine the correlations between significant SNPs and clinical characteristics.

**Results:**

Only rs143384 in the 5′UTR of *GDF5* was identified as significantly associated with increased susceptibility to chronic postsurgical pain, and the risk of A allele carriers was increased approximately 1.35-fold compared with that of G allele carriers. Haplotypes AGG and GGG in the LD block rs143384-rs224335-rs739329 also showed similar association patterns. Furthermore, we found that rs143384 was significantly correlated with chronic postsurgical pain in the subgroup aged ≤ 61 years, subgroup with a BMI ≤ 26, subgroup with no-smoking or no pain history, and subgroup with a drinking history.

**Conclusion:**

Our study provided supportive evidence that genetic variations in the *GDF5* gene are potential genetic factors that can increase the risk of chronic postsurgical pain in the Han Chinese population, but further research is necessary to elucidate the underlying mechanism.

**Supplementary Information:**

The online version contains supplementary material available at 10.1186/s13018-021-02549-5.

## Background

Pain is a universal feeling defined as an unpleasant sensory and emotional experience, and it varies considerably between populations for different environmental and genetic factors [1–3]. Although pain is a necessary warning signal of potential harm under certain conditions, chronic postsurgical pain (CPSP) is generally not conducive to postsurgical recovery. Pain is expected to be reduced with wound healing; however, some patients continue to have persistent pain known as CPSP, which affects 9.2 to 80.0% of surgery patients [4–6].

Chronic postsurgical pain is influenced by multiple physical factors, and considerable efforts have revealed that genetic variations are associated with the development of CPSP [7, 8]. Damage to neurological tissue during surgery is a prerequisite for CPSP, which elicits an excessive inflammatory response to regulate various inflammatory cytokines; the latter play key roles in the occurrence and maintenance of CPSP [9]. The growth differentiation factor 5 (*GDF5*) gene, located at 20q11.22 with 4 exons, encodes a secreted ligand of the TGF-β superfamily [10]. Its corresponding protein regulates the growth of neuronal axons and dendrites, as well as tissue development, including cartilage and the joint [11], and plays a role in the inflammatory response and tissue damage [12]. Additionally, genome-wide association studies (GWASs) have shown that the *GDF5* gene contributes to knee or hip osteoarthritis [11, 13, 14]. Therefore, we hypothesized that the *GDF5* gene is associated with CPSP, particularly in orthopedic surgery patients. The skeletal system supports body weight and movement, and studying the prognosis of orthopedic surgery is crucial to improve the daily life quality of patients. Single-nucleotide polymorphisms (SNPs) are common genomic DNA variations in populations, and SNPs located within functional regions of a gene may result in amino acid substitution and gene expression, which may be associated with susceptibility to diseases [15]. The rs143383 locus in the 5′UTR of *GDF5* is associated with increased susceptibility to osteoarthritis (including osteoarthritis of the knee, hip and hand) in both Asian and European populations [16–19], and different alleles of rs143383 influence *GDF5* gene expression in the whole joint, probably leading to increased susceptibility to osteoarthritis in individuals [20]. However, Shin et al. reported that rs143383 was not associated with primary knee osteoarthritis in a Korean population [21], and Tsuzou et al. reported a similar finding in Greek Caucasians of no significant differences in the subgroup stratified by sex [22]. Another two SNPs, rs224332 and rs224333, located in the *GDF5* gene were identified as related to hip dysplasia in Chinese women [23]. Knee pain is one of the most common musculoskeletal complaints that results in orthopedic surgery, although it is affected by various risk factors, such as sex, age, history of knee injuries, and smoking [24–26]. A recent genome-wide study by Meng et al. suggests that rs143384 in the *GDF5* gene is associated with knee pain in the UK Biobank, allele A is a risk factor [27], and SNP rs143384 in the 5′UTR region can affect *GDF5* expression [28], indicating that the *GDF5* gene is a potential risk factor for orthopedic CPSP.

However, the contribution of the *GDF5* gene to CPSP has not been directly elucidated based on biological evidence. Thus, exploring potential correlations between the *GDF5* gene and CPSP among different independent populations is necessary to enhance our understanding of the role of the *GDF5* gene in CPSP susceptibility. In the current study, we analyzed the genetic correlation of the *GDF5* gene with the risk of orthopedic CPSP and explored the risk factors correlated with CPSP in 3110 Han Chinese individuals. Our research aimed to uncover the relationship of SNP variants in the *GDF5* gene with orthopedic CPSP susceptibility and provide information to study the mechanisms involved in the etiology of CPSP.

## Materials and methods

### Subjects

In the present study, we recruited 1048 patients with CPSP as the case group and 2062 patients without CPSP as the control group from the First Affiliated Hospital of Xi’an Jiaotong University and Xi’an Honghui Hospital (all in Xi’an city). All the patients had undergone orthopedic surgery using total intravenous anesthesia; after discharge from the hospital, postsurgical pain was evaluated with cooperation. Patients who had previously undergone surgery, patients who abused pain medications, and patients with severe organic diseases were excluded from this study. All the recruited subjects were Han Chinese individuals. Additionally, to avoid potential population stratification in the study, the subjects who had an immigration history within three generations were not included in the present study. All the patients received patient-controlled intravenous analgesia (PCIA) 5 min before the surgical incision was closed. PCIA comprised fentanyl (15–20 mg/kg), dexamethasone 10 mg, and ondansetron 8 mg, which were diluted to 100 ml. During hospitalization, flurbiprofen axetil was injected intravenously as a combined analgesic to relieve inflammation (50 mg, twice a day). The same postsurgical pain management team used 11-point pain analog scale (PAS) to measure the severity of pain for all patients to complete the assessment of the pain severity over the surgical site that has persisted for 12 months after the operation. The severity score ranged from 0 to 10 (0 no pain, 10 worst imaginable pain). According to previous studies, we defined patients with a PAS score > 3 as the CPSP group [29]. Additionally, patients reported their current health status and pain management therapies including use of analgesics during the 12 months after the operation. Demographic and clinical information was collected from the study subjects (Table 1). All the subjects signed informed consent forms, and the study proposal was authorized by the ethics committee of the First Affiliated Hospital of Xi’an Jiaotong University.

**Table 1:** Basic characteristics of subjects

Variation	Case (*n* = 1048)	Control (*n* = 2062)	*P* value*
Age (year, mean ± SD)	61.1 ± 7.1	61.1 ± 7.6	0.95
Gender [n, (%)]			
Male	580 (55.3%)	1143 (55.4%)	0.98
Female	468 (44.7%)	919 (44.6%)	
BMI (mean ± SD)	25.97 ± 1.44	26.06 ± 1.56	0.11
PAS (mean ± SD)	2.27 ± 0.98 (0–3)	5.72 ± 1.60 (4–9)	***< 0.01***
Smoking [n, (%)]	250 (23.9%)	514 (24.9%)	0.51
Drinking [n, (%)]	311 (29.8%)	642 (31.1%)	0.40
With prior pain history	118 (11.3%)	220 (10.7%)	0.62

*PAS* pain analog scale, *SD* standard deviation

*Pearson χ^2^ value

### SNP selection and genotyping

To minimize the cost of the experiment while obtaining sufficient analysis data, we selected the tag SNPs in the *GDF5* gene for genotyping. First, we extracted all the SNPs with a minor allele frequency (MAF) ≥ 0.01 in the GDF5 gene. Next, we obtained all the tagged SNPs from the dataset of candidate SNPs based on the criterion of r^2^ ≥ 0.8. The Sequenom MassARRAY platform was used to genotype the 8 tag SNPs. The relevant information of these tag SNPs is shown in Supplementary Table S1. The genomic DNA used for genotyping came from the subject’s peripheral blood samples. A Tiangen DNA Extraction Kit was used for all DNA extraction according to the manufacturer’s instructions. In order to control the quality of genotyping, the technicians performing genotyping cannot know the case-control label of each sample in advance. In addition, we also conducted random sampling re-examinations on 5% of the samples. The re-examination results were completely consistent with the initial examination results.

### Statistical analyses

The association analysis of alleles and genotypes between the SNPs in the *GDF5* gene and orthopedic CPSP was performed using PLINK v1.9 software under three logistic models (additive, dominant and recessive), as well as the Hardy–Weinberg equilibrium (HWE) of all SNPs in the case and control groups. For allele association analysis, the odds ratio (OR) and 95% confidence interval (95% CI) were calculated to assess the association degree between SNPs in the *GDF5* gene and CPSP susceptibility. Statistical analysis for the basic parameters was implemented via Pearson chi-squared (χ^2^) test and unpaired Student’s *t* test using SPSS 19.0 (IBM Inc.). Haploview v4.2 was used to detect pairwise linkage disequilibrium (LD), and GENECOUNTING v2.2 was used to estimate the haplotype frequencies and association analysis by permutation testing. *P*< 0.05 was set as the threshold of statistically significant difference.

## Results

### Essential features of the participants

Table 1 demonstrates the essential features of the participants in our study. The case group contained 1048 CPSP individuals, and the control group of 2062 individuals without CPSP was similar in age, with mean ages of 61.1 ± 7.1 and 61.1 ± 7.6 years, respectively. In the case group, 44.7% of cases were male and 55.3% were female, and the corresponding percentages were 44.6% and 55.4% in the control group, respectively. The rates with a history of smoking, drinking, and prior pain in the case group vs control group were 23.9% vs 24.9%, 29.8% vs 31.1% and 11.3% vs 10.7%, respectively. Statistical analysis suggested no significant differences in the distribution of age, gender, lifestyles (smoking or drinking), or pain history (*P* > 0.05) between the two groups. Pain severity measured by PAS is an important component of the CPSP diagnosis, which is set as 11 grades from 0 to 10 (0 no pain, 10 worst imaginable pain); thus, the case group included individuals with scores of 4–10, and the control group included individuals with scores of 0–3. The PAS scores were statistically significant between the two groups (*P* < 0.001).

### Single SNP analysis between the GDF5 gene and CPSP risk

The allelic and genotypic frequency distributions of the 8 SNPs in the *GDF5* gene are shown in Table 2 and Supplemental Table S2. All 8 selected SNPs in the *GDF5* gene were in HWE in the case and control groups (*P* > 0.05), while the single SNP association analyses indicated that only rs143384 (G/A) was significantly different between the case and control groups. The minor allele G frequencies were 18.75% and 23.69% in the case and control groups, respectively, while the CPSP risk was increased in subjects with the A allele of rs143384 compared with that in G allele carriers (OR = 1.35; 95% CI 1.18–1.54; *P* = 7.85E−06), indicating that the A allele at rs143384 was a risk factor for CPSP. Moreover, the genotype frequency was 3.24% for GG, 31.01% for GA, and 65.75% for AA in the case group and 5.29% for GG, 36.81% for GA, and 57.90% for AA in the control group. The genotype frequencies of rs143384 were significantly different between the CPSP group and no-CPSP group under the three models (*P* = 3.92E−05, 2.32E−05, and 0.011 for the additive, dominant, and recessive models, respectively). Thus, genotypic analyses confirmed association signals similar to those of allelic analyses (Table 2).

**Table 2:** Multiple comparisons for correlation between *GDF* gene SNP rs143384 and CPSP risk

	Case (n = 1048)	Control (n = 2062)	P*
Gender: male			
GG	20 (1.91%)	63 (3.06%)	0.06
GA/AA	560 (53.43%)	1079 (52.32%)	
Female			
GG	14 (1.34%)	46 (2.23%)	0.08
GA/AA	454 (43.32%)	874 (42.39%)	
Age: ≤ 61 years			
GG	12 (1.15%)	48 (2.33%)	***0.02***
GA/AA	432 (41.22%)	816 (39.57%)	
> 61 years			
GG	22 (2.10%)	61 (2.96%)	0.17
GA/AA	582 (55.53%)	1137 (55.14%)	
BMI: ≤ 26			
GG	15 (1.43%)	56 (2.72%)	***0.02***
GA/AA	464 (44.27%)	854 (41.42%)	
> 26			
GG	19 (1.81%)	53 (2.57%)	0.22
GA/AA	550 (52.48%)	1099 (53.30%)	
Lifestyle: smoking			
GG	8 (0.76%)	29 (1.41%)	0.14
GA/AA	242 (23.09%)	485 (23.52%)	
No-smoking			
GG	26 (2.48%)	80 (3.88%)	***0.03***
GA/AA	772 (73.66%)	1468 (71.19%)	
Drinking			
GG	8 (0.76%)	37 (1.79%)	***0.03***
GA/AA	303 (28.91%)	605 (29.34%)	
No-drinking			
GG	26 (2.48%)	72 (3.49%)	0.13
GA/AA	711 (67.84%)	1348 (65.37%)	
Prior pain history			
GG	2 (0.19%)	9 (0.44%)	0.24
GA/AA	116 (11.07%)	211 (10.23%)	
No prior pain history			
GG	32 (3.05%)	100 (4.85%)	***0.02***
GA/AA	898 (85.69%)	1742 (84.48%)	

*P value was calculated by chi-square test; the significant value was shown in bold italics

**Table 3:** The results of single SNP association analyses of rs143384 and CPSP risk

Group	HWE *P*	Allele number (%)		Allelic P*	Genotype number (&)			Genotypic P*		OR^**^ (95% CI)
**rs143384**		G	A		GG	GA	AA	Additive	***3.92E−05***	
Case (*n* = 1048)	0.61	393 (18.75%)	1703 (81.25%)	***7.85E−06***	34 (3.24%)	325 (31.01%)	689 (65.75%)	Dominant	***2.32E−05***	1.35
Control (*n* = 2062)	0.43	977 (23.69%)	3147 (76.31%)		109 (5.29%)	759 (36.81%)	1194 (57.90%)	Recessive	***0.011***	(1.18–1.54)

*HWE* Hardy-Weinberg equilibrium, *OR* odds ratio, *CI* confidence interval

*The significant *P* value was shown in bold italics; ^**^OR is for the risk allele A of rs143384

### Stratified analyses between rs143384 and CPSP risk

We also evaluated the correlation between the *GDF5* gene SNP rs143384 and CPSP risk in different subgroups (Table 3). No significant association was found between rs143384 GG *vs* GA&AA and CPSP risk for the male and female subgroups (*P* > 0.05). Regarding age stratification, rs143384 GG *vs* GA&AA was significantly associated with CPSP risk for those aged ≤ 61 years (*P* = 0.02), and no significant difference was observed in the genotype distribution within the advanced age subgroup (> 61 years). In the subgroup with BMI ≤ 26, the genotype frequencies of rs143384 were significantly different between the case and corresponding control samples (*P* = 0.02), and similar results were found in the no-smoking subgroup, drinking subgroup, and subgroup without a pain history. We also explored the relationship between rs143384 and the PAS score in our samples. The genotype distribution of rs143384 was associated with different PAS scores (Table 4; *P* = 0.0162).

**Table 4:** The correlation between ***GDF5*** **gene** SNP rs143384 genotypes and PAS score

NRS	Genotype		P*
	GA/AA	GG	
0	179 (5.76%)	22 (0.71%)	***0.0162***
1	165 (5.31%)	23 (0.74%)	
2	496 (15.95%)	32 (1.03%)	
3	1113 (35.79%)	32 (1.03%)	
4	268 (8.62%)	26 (0.84%)	
5	278 (8.94%)	1 (0.03%)	
6	176 (5.66%)	1 (0.03%)	
7	105 (3.38%)	1 (0.03%)	
8	103 (3.31%)	1 (0.03%)	
9	84 (2.70%)	4 (0.13%)	

*P value was calculated by Student’s *t* test; the significant value was shown in bold italics

### Haplotype-based analyses

**Fig. 1:**
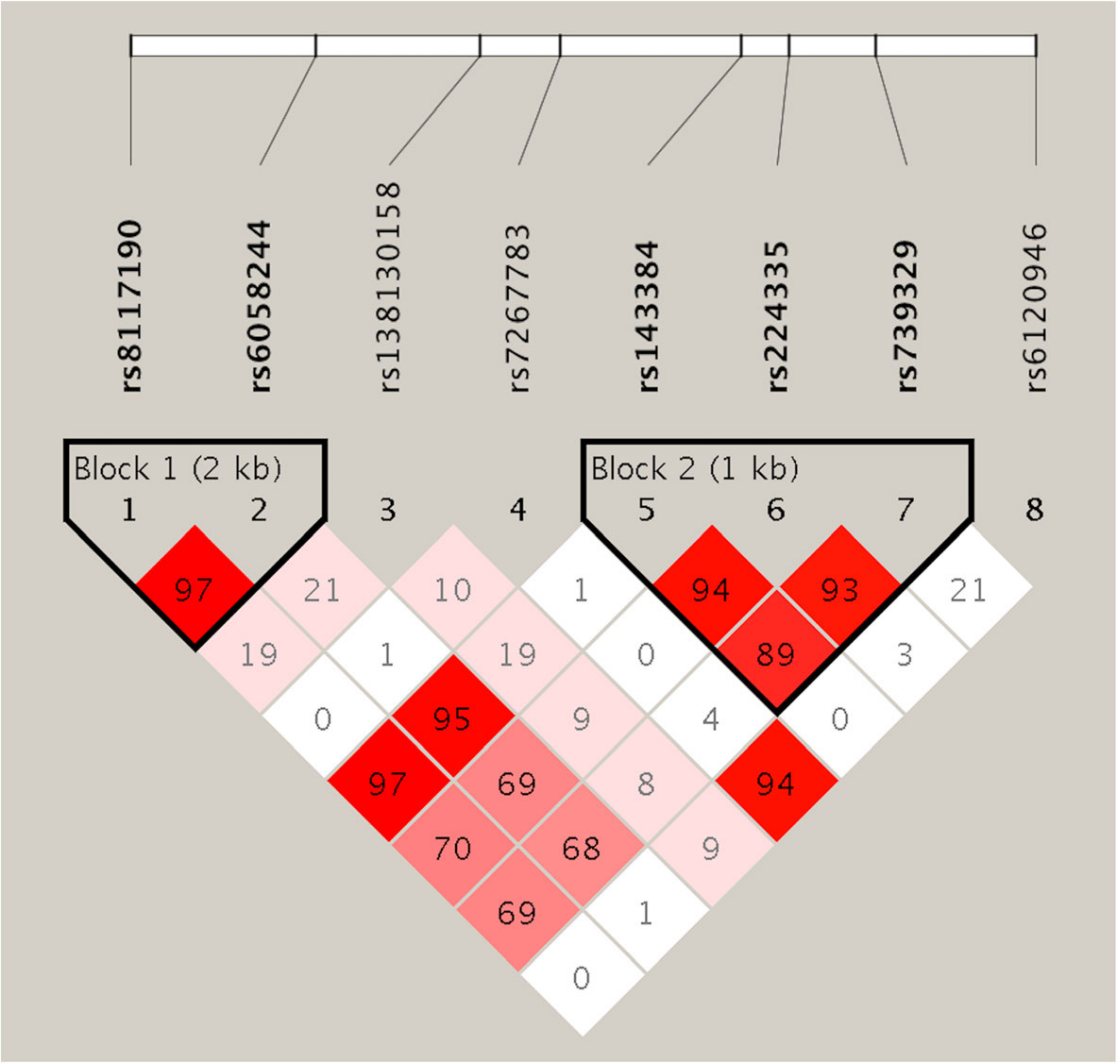
The linkage disequilibrium (LD) among 8 SNPs in GDF5 gene. Values of D′ are indicated in each cell

The 8 tag SNPs selected in this study map a 12.7-kb genomic region; hence, we examined the LD structure of these loci to perform haplotype-based association analyses. Two strong LD blocks (block 1: rs8117190-rs6058244; block 2: rs143384-rs224335-rs739329) were identified (Fig. 1), haplotypic associations of block 2 were observed with global *P < 0.000001*, four haplotypes were found in block 2 (Table 5), and the haplotypes AGG and GGG showed significant differences between the CPSP and control groups (*P* =  0.00012 and 0.00010, respectively).

**Table 5:** Haplotype-based association analyses in the study

Haplotype	Estimated frequencies (%)			Global *P* value**
	Case	Control	*P* value*	
rs143384-rs224335-rs739329				***< 0.000001***
AGG	80.27	75.95	***0.00012***	
GGG	11.61	15.24	***0.00010***	
GAA	3.78	4.48	0.19540	
GAG	3.32	3.88	0.26244	

Significant *P* values are in bold italics. Haplotypes are not shown, if frequency less than 1%

*Means based on 100,000 permutations

**Means based on comparison of frequency distribution of all haplotypes

## Discussion

Numerous studies have focused on the effect of gene polymorphisms on complex diseases in recent years and have shown that the *GDF5* gene contributes to various osteoarthritis forms [11, 13, 14]. Studies have proven that many pathological changes in osteoarthritis result from chronic low-grade inflammation mediated by inflammatory cytokines such as TNF-α and IL-1β [30]. Decreased *GDF5* expression in cartilage could lead to chronic arthritis in TNF-transgenic mice, whereas inflammatory conditions might influence *GDF5* expression via fibroblasts (inflammatory infiltration indicator) in osteoarthritis [31]. Therefore, we reasonably assumed that the *GDF5* gene is potentially related to orthopedic CPSP.

A previous study by Zhang et al. suggests that *GDF5* gene polymorphism is associated with knee osteoarthritis, and its interactions with age, BMI, and a history of drinking increase the risk [32]. Recently, a GWAS of knee pain-related genes identified associations with rs143384 located in the *GDF5* gene in the UK Biobank and identified the A allele as a risk factor [27]. The present study selected 8 tag SNPs in the *GDF5* gene and found that the *GDF5* gene is associated with orthopedic CPSP, among which the A allele of rs143384 in the 5′UTR of the *GDF5* gene could significantly increase the CPSP risk. Our study showed the first investigation of the correlation between *GDF5* and orthopedic CPSP in a Han Chinese population and suggested that interactions between the *GDF5* gene and age, BMI, and drinking history increased orthopedic CPSP susceptibility. SNP rs143384 in the *GDF5* gene was associated with CPSP in subsets aged ≤ 61 years, while Zhang et al. found significant differences in the *GDF5* gene polymorphism between knee osteoarthritis and the corresponding control group aged > 60 years in the Asian population [32]. This inconsistency suggests that the samples should be further investigated by stratification according to the type of surgery. Upregulated *GDF5* gene expression was observed in brown adipose tissues in obese mice, and its overexpression led to increased systemic energy expenditure [33], while the association between rs143384 and CPSP was only observed in subgroups with a BMI ≤ 26. Although smoking history and pain history had no interaction with the *GDF5* gene to affect orthopedic CPSP, a history of drinking did interact with the *GDF5* gene to associate with orthopedic CPSP. The reason may be that alcohol affects nervous system sensitivity, while *GDF5* gene expression is related to nerve synapses [12]. Furthermore, haplotype analyses in our study suggested that SNP rs143384 in *GDF5* was significantly associated with CPSP susceptibility, and both haplotypes AGG and GGG (rs143384-rs224335-rs739329) were associated with CPSP risk.

Although few reports have investigated the association between the *GDF5* gene and orthopedic CPSP, *GDF5* gene expression and polymorphic markers affecting osteoarthritis pain, particularly joint pain, have been published in humans from different ethnic groups. In particular, the SNP rs143383 (T/C) in the 5′UTR of the *GDF5* gene is a major susceptibility factor for osteoarthritis in Asian and European populations [17, 19, 34]. The allelic expression of rs143383 can lead to reduced *GDF5* expression because the CpG sites formed by allele C of rs143383 are methylated in cells and joint tissues, and *GDF5* is critical for joint homeostasis [20, 35]. Using luciferase reporter assays, Egli et al. suggest that the rs143384 SNP in the 5′UTR of the GDF5 gene interacts with rs143383 in vitro [28]. It is reasonable to believe that rs143384 may affect *GDF5* gene expression and is associated with osteoarthritis and orthopedic CPSP. *GDF5* encodes a secreted ligand of the TGF-β superfamily [10] that regulates the growth of neuronal axons and dendrites, as well as tissue development, including cartilage and joints [11], and plays a role in the inflammatory response and tissue damage [12]. Functional studies suggest that GDF5 absence in mouse models profoundly affects knee morphology [36, 37]. As a functional protein, GDF5 supplementation has therapeutic potential in the chondrogenic process and maintenance of cartilage homeostasis [38]. Degenkolbe et al. indicate that the superagonistic *GDF5* variant shows faster and more efficient bone defect healing in patients with multiple synostoses syndrome using an animal model [39]. Given that different *GDF5* pathogenic mutations are related to different clinical features, the depression of *GDF5* may result in bone development defects, and overexpression causes excessive bone formation [40]. The current study suggests that SNP rsl43384 in *GDF5* is associated with orthopedic CPSP. The underlying mechanism may be that *GDF5* gene expression influences the rate of healing of injured tissue after orthopedic surgery, and a proper healing rate usually reduces postsurgical pain. Although our study is based on a moderate sample size, some deficiencies should not be ignored. In particular, stratified analysis must be confirmed in other ethnic populations. Additionally, our study lacks functional data to elucidate the underlying interaction mechanisms between SNP rs143384 in *GDF5* and orthopedic CPSP. Most importantly, the clinical symptoms of our samples should be considered to refine the correlation between rs143384 and the phenotype to determine the potential of these biomarkers in clinical applications.

Generally, our present findings, through several sets of analyses, suggest that rs143384 in the *GDF5* gene is strongly associated with CPSP in the Han Chinese population and is a potential gene for CPSP sensitivity. However, the mechanism by which *GDF5* affects CPSP remains unclear. In-depth investigation of the pathological mechanisms and fundamental role of *GDF5* in CPSP is necessary and will facilitate potential clinical applications.

## Supplementary Information

**Additional file 1: Supplemental Table S1**. Basic information of the SNPs selected for genotyping. **Supplemental Table S2**. The results of single SNP-based association analyses of the rest 7 SNPs.

## Data Availability

Please contact the authors for reasonable requests.

## Abbreviations

GDF5: Growth differentiation factor 5
CPSP: Chronic postsurgical pain
GWAS: Genome-wide association study
SNP: Single nucleotide polymorphism
PCIA: Patient-controlled intravenous analgesia
PAS: Pain analog scale
MAF: Minor allele frequency
HWE: Hardy-Weinberg equilibrium
LD: Linkage disequilibrium
ORs: Odds ratios
CI: Confidence interval
