# Supplementary material for: The effect of common variants in GDF5 gene on the susceptibility to chronic postsurgical pain
Source: J Orthop Surg Res. 2021 Jul 1;16:420. doi: 10.1186/s13018-021-02549-5 (PMC8247225; doi:10.1186/s13018-021-02549-5)
Supplement: Supplementary file 1 — Additional file 1: Supplemental Table S1. Basic information of the SNPs selected for genotyping. Supplemental Table S2. The results of single SNP-based association analyses of the rest 7 SNPs. [file 13018_2021_2549_MOESM1_ESM.docx]

Supplemental Table S1. Basic information of the SNPs selected for genotyping

| CHR | BP | SNP | A1 | FUNCTION |
| --- | --- | --- | --- | --- |
| 20 | 34017186 | rs8117190 | C | None |
| 20 | 34019788 | rs6058244 | C | Upstream transcript variant |
| 20 | 34022085 | rs138130158 | A | Missense variant |
| 20 | 34023219 | rs7267783 | T | Intron variant |
| 20 | 34025756 | rs143384 | G | 5’ upstream transcript variant |
| 20 | 34026422 | rs224335 | A | Intron variant |
| 20 | 34027636 | rs739329 | A | Intron variant |
| 20 | 34029882 | rs6120946 | T | Intron variant |

CHR: chromosome; BP: position of base pair; A1: minor allele

Supplemental Table S2. The results of single SNP-based association analyses of the rest 7 SNPs.

| SNP loci | Genotype frequency (%) | | | HWE *P* | Genotypic *P** | Allele frequency (%) | | Allelic *P* |
| --- | --- | --- | --- | --- | --- | --- | --- | --- |
| **rs8117190** | CC | CT | TT |  |  | C | T |  |
| Case (n=1048) | 27(2.6%) | 302(28.8%) | 719(68.6%) | 0.5834  0.6925 | 0.7063 | 356(17.0%) | 1740(83.0%) | 0.8017 |
| Control (n=2062) | 60(2.9%) | 569(27.6%) | 1433(69.5%) |  |  | 689(16.7%) | 3435(83.3%) |  |
| **rs6058244** | CC | CT | TT |  |  | C | T |  |
| Case (n=1048) | 30(2.9%) | 293(28.0%) | 725(69.2%) | 0.9125 | 0.6426 | 353(16.8%) | 1743(83.2%) |  |
| Control (n=2062) | 48(2.3%) | 573(27.8%) | 1441(69.9%) | 0.3318 |  | 669(16.2%) | 3455(83.8%) | 0.5384 |
| **rs138130158** | AA | AC | CC |  |  | A | C |  |
| Case (n=1048) | 8(0.8%) | 128(12.2%) | 912(87.0%) | 0.1435 | 0.6614 | 144(6.9%) | 1952(93.1%) |  |
| Control (n=2062) | 11(0.5%) | 243(11.8%) | 1808(87.7%) | 0.3569 |  | 265(6.4%) | 3859(93.6%) | 0.5162 |
| **rs7267783** | TT | TC | CC |  |  | T | C |  |
| Case (n=1048) | 6(0.6%) | 128(12.2%) | 914(87.2%) | 0.4565 | 0.8157 | 140(6.7%) | 1956(93.3%) |  |
| Control (n=2062) | 14(0.7%) | 268(13.0%) | 1780(86.3%) | 0.2474 |  | 296(7.2%) | 3828(92.8%) | 0.4947 |
| **rs224335** | AA | AG | GG |  |  | A | G |  |
| Case (n=1048) | 9(0.9%) | 143(13.6%) | 896(85.5%) | 0.1951 | 0.3369 | 161(7.7%) | 1935(92.3%) |  |
| Control (n=2062) | 16(0.8%) | 322(15.6%) | 1724(83.6%) | 0.7791 |  | 354(8.6%) | 3770(91.4%) | 0.2427 |
| **rs739329** | AA | AG | GG |  |  | A | G |  |
| Case (n=1048) | 4(0.4%) | 86(8.2%) | 958(91.4%) | 0.1506 | 0.6659 | 94(4.5%) | 2002(95.5%) |  |
| Control (n=2062) | 6(0.3%) | 186(9.0%) | 1870(90.7%) | 0.4705 |  | 198(4.8%) | 3926(95.2%) | 0.6121 |
| **rs6120946** | TT | TA | AA |  |  | T | A |  |
| Case (n=1048) | 17(1.6%) | 247(23.6%) | 784(74.8%) | 0.6915 | 0.7272 | 281(13.4%) | 1815(86.6%) |  |
| Control (n=2062) | 37(1.8%) | 511(24.8%) | 1514(73.4%) | 0.4693 |  | 585(14.2%) | 3539(85.8%) | 0.416 |

* *P* value was calculated under additive model.
